# Discovery of Novel Protein-Coding and Long Non-coding Transcripts in Distinct Regions of the Human Brain

**DOI:** 10.1007/s12031-025-02316-9

**Published:** 2025-03-06

**Authors:** Kristina Santucci, Yuning Cheng, Si-Mei Xu, Yulan Gao, Grace Lindner, Konii Takenaka, Michael Janitz

**Affiliations:** School of Biotechnology and Biomolecular Sciences, University of New South Wales, Sydney, Australia

**Keywords:** Alternative splicing, Long-read sequencing, Transcript isoforms, Novel isoforms, Brain, Transcriptomics

## Abstract

Recent improvements in the accuracy of long-read sequencing (LRS) technologies have expanded the scope for novel transcriptional isoform discovery. Additionally, these advancements have improved the precision of transcript quantification, enabling a more accurate reconstruction of complex splicing patterns and transcriptomes. Thus, this project aims to take advantage of these analytical developments for the discovery and analysis of RNA isoforms in the human brain. A set of novel transcript isoforms was compiled using three bioinformatic tools, quantifying their expression across eight replicates of the cerebellar hemisphere, five replicates of the frontal cortex, and six replicates of the putamen. By taking a subset of the novel isoforms consistent across all discovery methods, a set of 170 highly confident novel RNA isoforms was curated for downstream analysis. This set consisted of 104 messenger RNAs (mRNAs) and 66 long non-coding RNAs (lncRNAs) isoforms. The detailed structure, expression, and potential encoded proteins of novel mRNA isoform BambuTx321 have been further described as an exemplary representative. Additionally, the tissue-specific expression [mean counts per million (CPM) of 5.979] of novel lncRNA, BambuTx1299, in the cerebellar hemisphere was observed. Overall, this project has identified and annotated several novel RNA isoforms across diverse tissues of the human brain, providing insights into their expression patterns and investigating their potential functional roles. Thus, this project has contributed to a more comprehensive understanding of the brain’s transcriptomic landscape for applications in basic research.

**Supplementary Information:**

The online version contains supplementary material available at 10.1007/s12031-025-02316-9.

## Introduction

Alternative splicing (AS) of precursor mRNA (pre-mRNA) allows for the generation of multiple RNA isoforms from the same gene. This cellular process expands the functional eukaryotic proteome with a low number of protein-coding genes (Nilsen & Graveley 2010). Recent estimates suggest that AS occurs for more than 95% of multi-exonic human genes, which places this cellular mechanism at the forefront of regulating post-transcriptional gene expression (Jiang & Chen 2021; Lee & Rio 2015). AS forms mRNA transcripts with distinct features from the constitutive sequence, which show differential and specific expression in different tissues, during different stages of development, and in diseased states (Marasco & Kornblihtt 2023). The human brain has an elaborate transcriptomic architecture encompassing the expression of more than 76% of protein-coding genes (Sjöstedt et al. 2020). The RNA landscapes of different tissues within the brain are heterogeneous, with a high number of tissue-specific long non-coding and protein-coding transcripts in comparison to other organs (Zhu et al. 2016).

Short-read RNA sequencing (RNA-seq) technologies are invariably limited in their ability to capture such transcriptomic complexity, hindered by the requirement of RNA fragmentation during library preparation. Although short-read sequencing (SRS) can cover individual splice junctions with high accuracy, the task of unambiguously assigning such reads to isoforms with similar structures is challenging, in terms of both assembly and quantification. Long-read sequencing (LRS) experiments have demonstrated the potential to overcome these challenges, as longer read lengths implicate greater coverage of multiple informative splice junctions. This reduces the uncertainty in assigning reads to isoform models, making it easier to capture the complete transcriptomic composition of a sample (De Paoli-Iseppi et al. 2021). There are multiple computational tools available for the detection of novel transcript isoforms in LRS data, outlined by Santucci et al. (2024). Most tools can quantify the expression of both annotated and unannotated isoforms within the sample (Santucci et al. 2024). Utilising such tools has already demonstrated that LRS can detect novel isoforms previously unresolved by SRS studies (Chen et al. 2023; Cole et al. 2020; Dana et al. 2020; Fang et al. 2021; Gao et al. 2023; Zhang et al. 2023, 2020; Zhou et al. 2023). However, LRS technologies such as nanopore sequencing have limitations that must be considered when assembling transcripts. In comparison to second-generation RNA-seq technologies, early nanopore sequencing technologies were less accurate due to base-calling errors. Notably, this accuracy is now comparable to SRS technologies in the most recent kits. Despite this, read alignment programs such as Minimap2 that use seed-and-extend algorithms have a fall in accuracy at long tandem repeats and at small exons (< 40 bps) because of the lack of seeds at these regions (Liu et al. 2023). High sequencing error rates are particularly prevalent at splice junctions, which compromises their alignment (Liu et al. 2019; Mikheenko et al. 2022; Parker et al. 2021).

Overall, transcriptome assemblies from LRS enable the analysis of biologically relevant AS events. The recent and numerous developments in bioinformatic tools for LRS have demonstrated the ability to accurately detect and quantify new RNA isoforms, in both poor and well-annotated species (Santucci et al. 2024). For example, long-read RNA sequencing (RNA-seq) data of prised Genotype-Tissue Expression (GTEx) project samples was recently produced (Glinos et al. 2022). The study by Glinos et al. (2022) uncovered upwards of 70,000 new transcriptional isoforms across various tissues of the human body not previously resolved by short-read RNA-seq data. However, recent findings from the Long-read RNA-Seq Genome Annotation Assessment Project (LRGASP) proposed that to accurately resolve novel isoforms, more than one transcript assembly algorithm should be utilised, as well as using high-quality data with replicates. Taking these findings and recommendations into consideration, we demonstrated the ability to curate novel transcripts detected by three different assembly tools, discovering and annotating novel transcriptional isoforms in various regions of the human brain. Here, we explore both protein-coding and non-coding novel RNA isoforms across the cerebellar hemisphere, frontal cortex, and the putamen utilising the LRS data produced by Glinos et al. (2022). As some RNA isoforms show specificity in expression at both the tissue and region level (Hu et al. 2023; Leung et al. 2021; Shimada et al. 2024; Zhu et al. 2016), this study also investigates the expression patterns of novel mRNA and lncRNA isoforms.

Overall, by integrating recent machine learning (ML) refinements and rule-based filtering techniques, this study intends to explore and provide a more comprehensive understanding of the transcriptomic diversity in the human brain. As a result of this analysis, we seek to provide a robust reference dataset derived from healthy brain samples. This dataset can serve as a valuable baseline for future comparative studies, facilitating the identification of transcriptional alterations associated with various brain diseases and disorders.

## Materials and Methods

### Acquisition of Sequencing Data

Raw long-read RNA-seq data was accessed from the GTEx v9 database under the National Centre for Biotechnology Information (NCBI) database of Genotypes and Phenotypes (dbGaP) accession number phs000424.v9 (GTEx Consortium 2013, 2020; Tryka et al. 2014). The sequencing data was generated by Glinos et al. (2022) and is derived from tissue samples taken from the GTEx project ("The Genotype-Tissue Expression (GTEx) project," 2013; Glinos et al. 2022; GTEx Consortium 2020). The criteria for donor inclusion are detailed by the GTEx Consortium (2020), but male and female donors (Table S1) were selected between ages 21 and 70 (inclusive) with less than 24 h between the time of death and harvesting of tissues, which were preserved in PAXgene® tissue kits (PreAnalytiX®) (Carithers et al. 2015). A total of 19 brain samples from ten male and six female donors were utilised in this study. These samples are derived from three distinct regions of the brain: the frontal cortex (BA9) (*n* = 5), cerebellar hemisphere (*n* = 8), and putamen (*n* = 6) (Table S1).

A detailed methodology for the tissue preparation and sequencing protocol is described by Glinos et al. (2022). In brief, total RNA was extracted and isolated from the PAXgene® fixed samples with the Qiagen® PAXgene® Tissue miRNA kit at the BROAD Institute of MIT and Harvard as described by Carithers et al. (2015) and Glinos et al. (2022). The isolated RNA was reverse-transcribed into cDNA before polymerase chain reaction (PCR) amplification. The samples were then sequenced on Oxford Nanopore Technology (ONT) MinION and GridION X5 platforms for 48 h following a cDNA-PCR protocol outlined by Glinos et al. (2022). Finally, the raw signals were base-called with Guppy v3.2.4 (ONT).

Downstream analysis of select novel transcripts was validated with an independent short-read RNA-seq dataset provided by GTEx Consortium (2020). The detailed methodology for RNA sequencing is described by GTEx Consortium (2020) but in brief, sequencing was performed using the Illumina TruSeq™ RNA sample preparation protocol. This method used polyA tail selection with oligo dT beads and was not strand-specific. The raw sequencing reads were aligned to the Genome Reference Consortium Human Build 38 (GRCh38) with STAR v2.5.3a (Dobin et al. 2013) as described by GTEx Consortium (2020).

### Long-Read RNA-Seq Data Pre-processing and Two-Pass Read Alignment

**Fig. 1:**
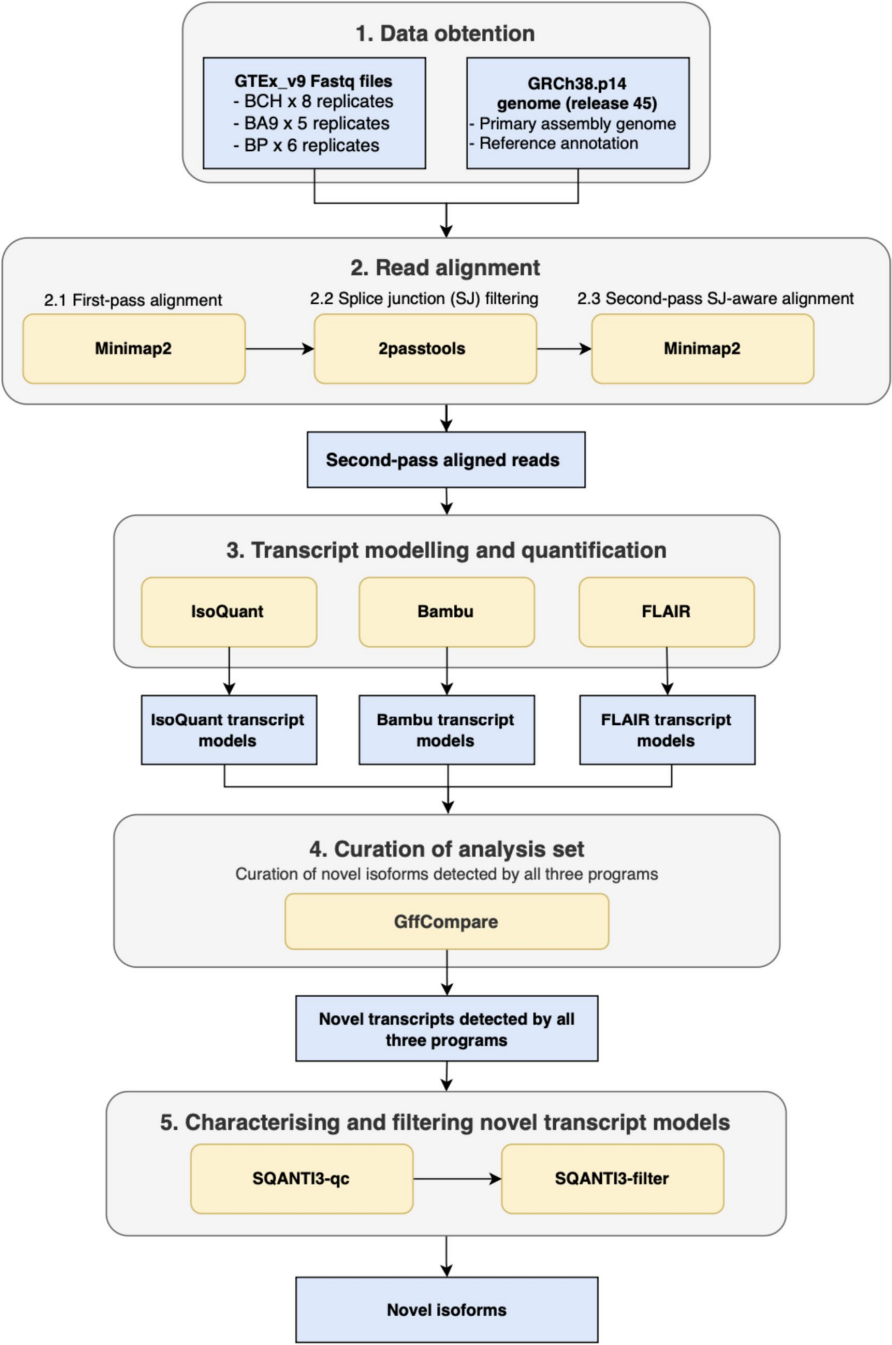
Analytical pipeline. Bioinformatic workflow for data pre-processing, transcript modelling, and the curation of novel isoforms. Abbreviations: BA9, Brodmann area 9 (frontal cortex); BCH, cerebellar hemisphere; BP, putamen; GRCh38.p14, Genome Reference Consortium Human Build 38 patch 14

A simplified schematic of the studies’ analytical pipeline is outlined (Fig. 1). All analysis code and scripts have been made available on GitHub (see “Code Availability”) which utilise publicly available packages. Their full program codes are listed and described by the respective references in Table S2. The long-read RNA-seq reads were aligned to the GRCh38 patch 14 (GRCh38.p14) genome (GENCODE version 45) in a two-pass approach with Minimap2 v2.24 (Li 2018). First-pass alignment was performed using the tags *-a –cs* = *long -k14 -x splice*. Splice junctions from this first-pass alignment were then scored with 2passtools v0.3.1 (Parker et al. 2021), based on junction metrics and sequence information extracted with an ML logistic regression model. The low scoring splice junctions were filtered. For the second-pass alignment, reads were aligned with Minimap2 with the same tags as the first-pass alignment, with the addition of the 2passtools filtered junctions supplied with the *–junc-bed* flag for a splice junction-guided second-pass alignment.

### Transcriptome Assembly and Isoform Quantification

Transcript models were assembled using Bambu v3.6.0 (Chen et al. 2023), IsoQuant v3.4.2 (Prjibelski et al. 2023), and FLAIR v2.0.0 (Tang et al. 2020) from second-pass read alignments. Transcript modelling with Bambu was performed across the three brain regions simultaneously, quantifying the normalised expression for each replicate. Novel isoform discovery was conducted using a recommended Novel Discovery Rate (NDR) threshold of 0.152.

Prior to transcript assembly with FLAIR collapse, the FLAIR *correct* module was used to correct any misaligned splice sites as well as to supply the *collapse* module with the correctly formatted input files. For transcript assembly, the raw fastq reads were supplied with the parameter *–reads* alongside the recommended parameters *–stringent*, *–generate_map*, *–annotation_reliant generate*, and *–check_splice*. Assembled transcripts were quantified with the FLAIR *quantify* module using the same recommended parameters in the previous step.

IsoQuant was run in the default nanopore mode (*–data_type nanopore*). The expression across individual replicates was also determined by supplying a list of input files (aligned, sorted, and indexed bam files) to run concurrently with –*bam_list*.

A two-dimensional principal component analysis (PCA) was also performed on the Bambu transcript assembly. For each transcript, Bambu calculated the standard deviation (SD) of normalised expression values (CPM) across all samples, followed by the median SD. Transcripts with more variable expressions (SD > median SD) were used for PCA analysis. The CPM of such transcripts were log transformed (log2(CPM + 1)) for visualisation. Spearman’s Correlation Coefficient (SCC) was calculated between the log-transformed transcript expression values across all 19 samples for hierarchical clustering with Bambu. The plots from both PCA and hierarchical clustering analysis were generated with Bambu in an R environment (v4.40).

### Quality Control, Filtering, and Characterisation of Novel Transcripts

GffCompare v0.11.2 (Pertea & Pertea 2020) was used to curate a list of novel transcripts that were resolved by all three transcript assembly programs. The transcript models from Bambu, IsoQuant, and FLAIR were supplied to GffCompare in Gene Transfer Format (GTF) for transcript matching. As a result, a list of non-redundant transcripts was generated in a tracking file (.tracking). Novel transcripts present in and structurally equivalent across all three programs were extracted.

The transcript models from Bambu, IsoQuant, and FLAIR were characterised with the SQANTI3 v5.2.1 *quality control* module (Pardo-Palacios et al. 2024a, b). This module also identifies coding sequences (CDS) and predicts open reading frames (ORFs) with the GeneMarkS-T algorithm (Tang et al. 2015). The characterised transcripts were then filtered with the SQANTI3 *filter* module. A user-defined rules filter was supplied to the program to remove mono-exonic and artifactual transcripts; this filter was structured with three independent rules. All transcripts were removed if the 3′ end was flagged as a potential intra-priming event, indicated by the presence of 12 or more adenines in the 20 bps genomic window downstream of the Transcription Terminating Site (TSS). For full-splice matches, this was the only filter applied. For the remaining set of transcript structural categories, transcripts were filtered if they were mono-exonic or had a splice junction that was the result of a reverse transcriptase template switching (RT-switching) event. Additionally, all novel junctions must have had a short-read coverage above a threshold of three to have been retained. Finally, novel transcripts present in and structurally equivalent across the three programs that had also passed the rules filter were identified to form a refined set of novel isoforms. It should be noted that incomplete splice matches (ISMs) were not explored, as they could either represent alternative transcripts shorter at the 3′ and/or 5′ end or be the result of RNA degradation.

### Visualisation of Novel Isoforms

The structure and expression of novel isoforms were visualised against annotated isoforms at the same loci with Bambu v3.6.0 (Chen et al. 2023) in an R v4.40 environment. Expression values are base-2 logarithmic transformations of normalised transcript expression values (log2(CPM + 1)).

### Novel Protein-Coding Isoforms Analysis

ORFs of coding novel isoforms were compared to a database of protein sequences using the protein–protein Basic Local Alignment Search Tool (blastp) (Altschul et al. 1990) with the default search parameters. For sequences that had no homology to human proteins in the initial search, a second search was conducted expanding the maximum target sequences from 100 to 5000 bps, as well as filtering the non-redundant protein sequences database for only *Homo sapiens* (taxid:9606). Sequences with low or no homology to known human proteins were then scanned against the InterProScan database of signatures to identify protein domains (Quevillon et al. 2005). Conserved regions and domains were also determined by comparing to NCBI’s Conserved Domain Database with NCBI Conserved Domain Search.

ORF sequences of interest were uploaded to the AlphaFold 3 (Abramson et al. 2024) server to predict the protein structure of these novel protein sequences. The predicted models were uploaded to ChimeraX v1.8 for the visualisation of features such as alpha chains, beta sheets, peptide binding sites, and protein domains. The predicted local distance difference test (plDDT) and predicted aligned error (PAE) confidence scores of the models were also uploaded to ChimeraX to define PAE domains using the Croll TI PAE Graph Clustering Algorithm. This algorithm segments the model into regions of residues predicted to be positioned relative to one another, according to their PAE.

## Results

### Principal Component Analysis and Hierarchical Clustering

**Fig. 2:**
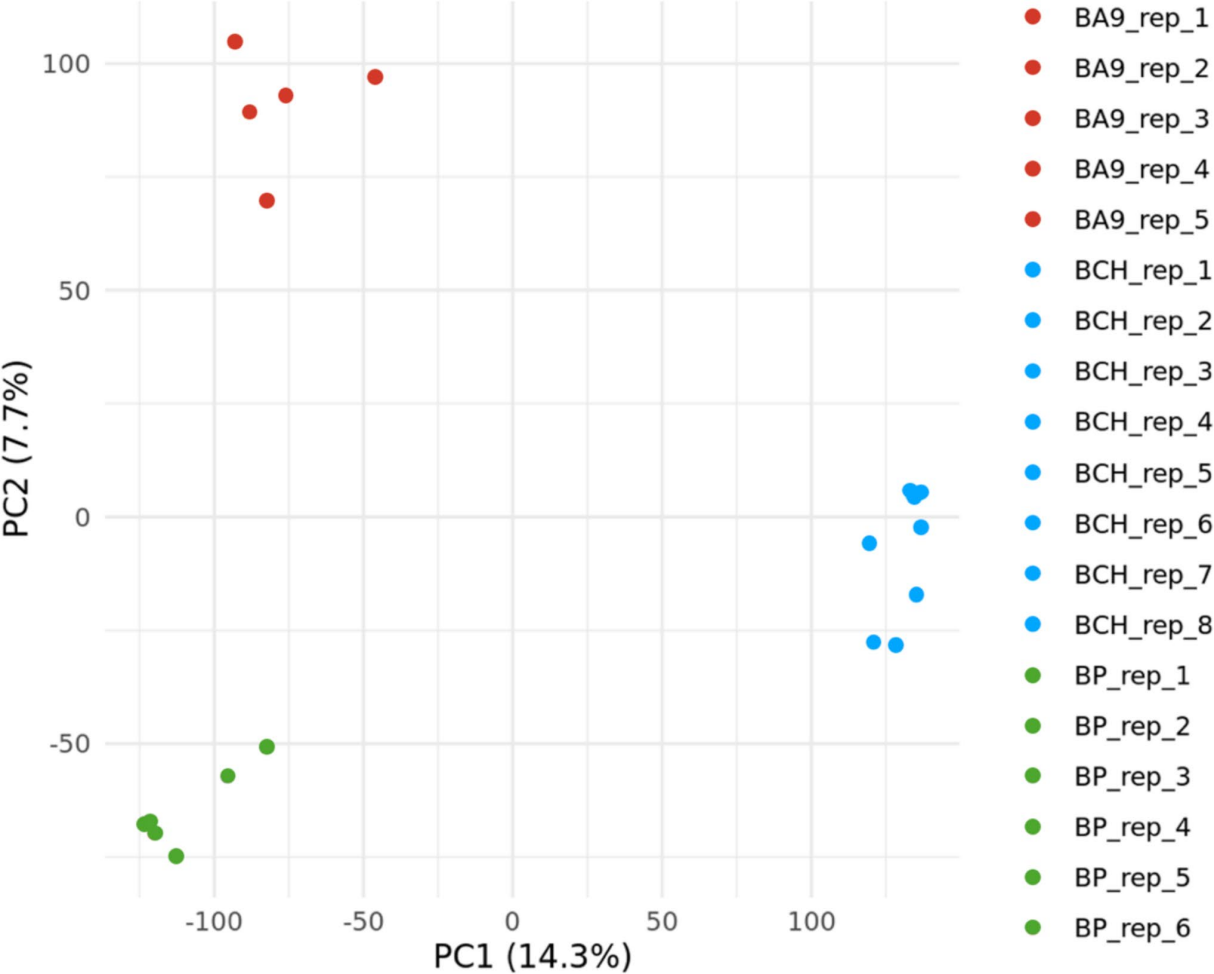
Principal component analysis of transcriptomic profiles in the human brain. A two-dimensional PCA was performed on the transcriptome assemblies of the frontal cortex (*n* = 5), cerebellar hemisphere (*n* = 8), and putamen (*n* = 6). Transcripts with variable expressions across the samples (SD > median SD) were used in the PCA. Expression values are base-2 logarithmic transformations of CPM values (log2(CPM + 1)). The percentage of total variation by PC1 and PC2 are shown on the x-axis and y-axis respectively. Abbreviations: PC1, principal component 1; PC2, principal component 2; BA9, Brodmann area 9 (frontal cortex); BCH, cerebellar hemisphere; BP, putamen

A two-dimensional PCA was performed to discern the variation in transcript expression across all samples from the putamen, cerebellar hemisphere, and frontal cortex. As seen in the PCA plot, the first two principal components account for 14.3% and 7.7% (respectively) of the variance in transcript expression between all samples (Fig. 2). PC1 and PC2 together account for 22% of the total variance, with replicates distinctly clustering according to their tissue of origin.

**Fig. 3:**
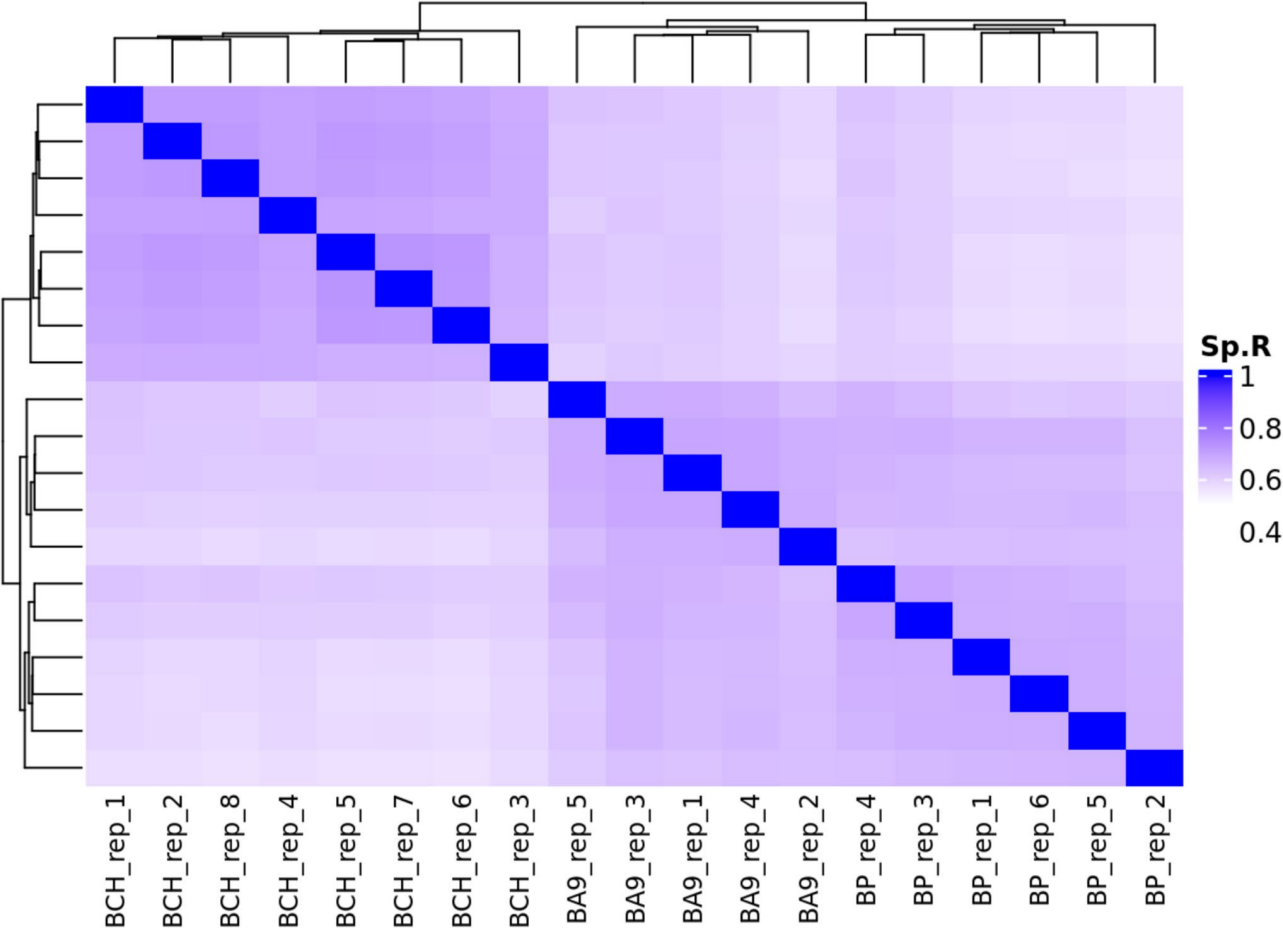
Hierarchical clustering of transcriptomes in the human brain. Hierarchical clustering of the transcriptomic profiles across the frontal cortex (*n* = 5), cerebellar hemisphere (*n* = 8), and the putamen (*n* = 6). Transcripts with variable expressions across the samples (SD > median SD) were used in the analysis. Correlation was calculated using Spearman’s correlation coefficient of transformed expression values (log2(CPM + 1)). The plots were generated with Bambu. v.3.6.0 in an R environment (v4.40). Abbreviations: Sp.R, Spearman’s rho; BCH, cerebellar hemisphere; BP, putamen; BA9, Brodmann area 9 (frontal cortex)

Hierarchical cluster analysis was performed on the transcript expression profiles of all replicates from the putamen (*n* = 6), frontal cortex (*n* = 5), and cerebellar hemisphere (*n* = 8). Clustering and the generation of a heatmap (Fig. 3) were performed with Bambu. v.3.6.0 in an R environment (v4.40). The correlation between samples was calculated using SCC of transformed transcript expression values (log2(CPM + 1)). Three moderately distinct clusters can be seen in the dendrogram, which correspond to the three brain regions from which the transcriptomic data was obtained (Fig. 3). In conjunction with the PCA analysis, a moderate similarity between the transcript profiles of the putamen, frontal cortex, and cerebellar hemisphere is shown. Samples from the putamen and the frontal cortex are more closely associated with each other compared to samples from the cerebellar hemisphere, suggesting the transcriptomic profiles of these two regions reflect more closely related biological functions (Fig. 3).

### Novel Isoform Discovery and Quantification

The mean number of transcripts assembled across all experimental replicates by Bambu, FLAIR, and IsoQuant was approximately 107,457, 216,237, and 71,260 respectively. However, over 73% of the mean total transcripts assembled by FLAIR were classed as artifactual. Notably, a large proportion of the filtered FLAIR transcripts were intergenic mono-exonic transcripts (73.52%) and hence were excluded from the filtered set by the rules filter. In fact, all genic-genomic, antisense, and intergenic transcripts assembled with FLAIR were classed as artifactual. IsoQuant assembled 21,534 novel transcripts that had passed the rules filter across all replicates (Table S3). The majority of these transcripts were expressed in all three regions (*n* = 13,500) whilst only 2,890 were expressed in only one of the three brain regions (Table S6). Similarly, FLAIR assembled 22,469 unannotated non-artifactual transcripts (Table S4), with 10,448 being expressed in all regions and 4340 being uniquely expressed in only one region (Table S7). Bambu had the highest number of full-splice matches, in other words assembled reference transcripts, with an average of 105,704 across all samples. Additionally, Bambu assembled 1792 novel transcripts that passed the filter (Table S5), with 1390 having expression in all three regions, and 139 in only one (Table S8). Comparably, over 70,000 novel transcripts were identified with FLAIR in the Glinos et al. (2022) study, which utilised 90 samples from 14 tissues from various systems of the human body. The lower yield of novel transcripts from Bambu assemblies is a result of the algorithm’s stringency, as an NDR threshold of 0.152 was used. The NDR is a transcript discovery parameter between 0 and 1 that represents the proportion of novel transcripts from the set of total transcripts that have an equal or higher transcript score. Under this definition, it is estimated that approximately 85% of all transcripts will be annotated and ~ 15% will be novel. This threshold essentially acts as an upper limit on the false positive rate, increasing the precision of novel isoform detection. A caveat to this stringency is the potential exclusion of true positive novel isoforms; however, analysing a high number of potentially false positive novel transcripts is also not suitable.

To further reduce the inclusion of false positive novel isoforms, a set of isoforms that were consistent in structure and expression across the three discovery methods (Bambu, IsoQuant, and FLAIR) was curated, all of which also passed the rules filter (Table S9). In total, there were 170 isoforms in the analysis set, 104 of which were determined to have protein-coding potential. There were 70 transcripts that were novel combinations of known splice junctions, 89 that were novel combinations of known splice sites, five that were novel by intron retention, and six that were novel fusion transcripts. Most of these novel isoforms were expressed across all three brain regions (*n* = 159) (Table S9). Seven novel isoforms from the analysis set were expressed in only the frontal cortex and putamen. Additionally, one isoform was uniquely expressed in the frontal cortex, and another uniquely expressed in the cerebellar hemisphere. No novel isoforms in the analysis set were uniquely expressed in the putamen.

**Fig. 4:**
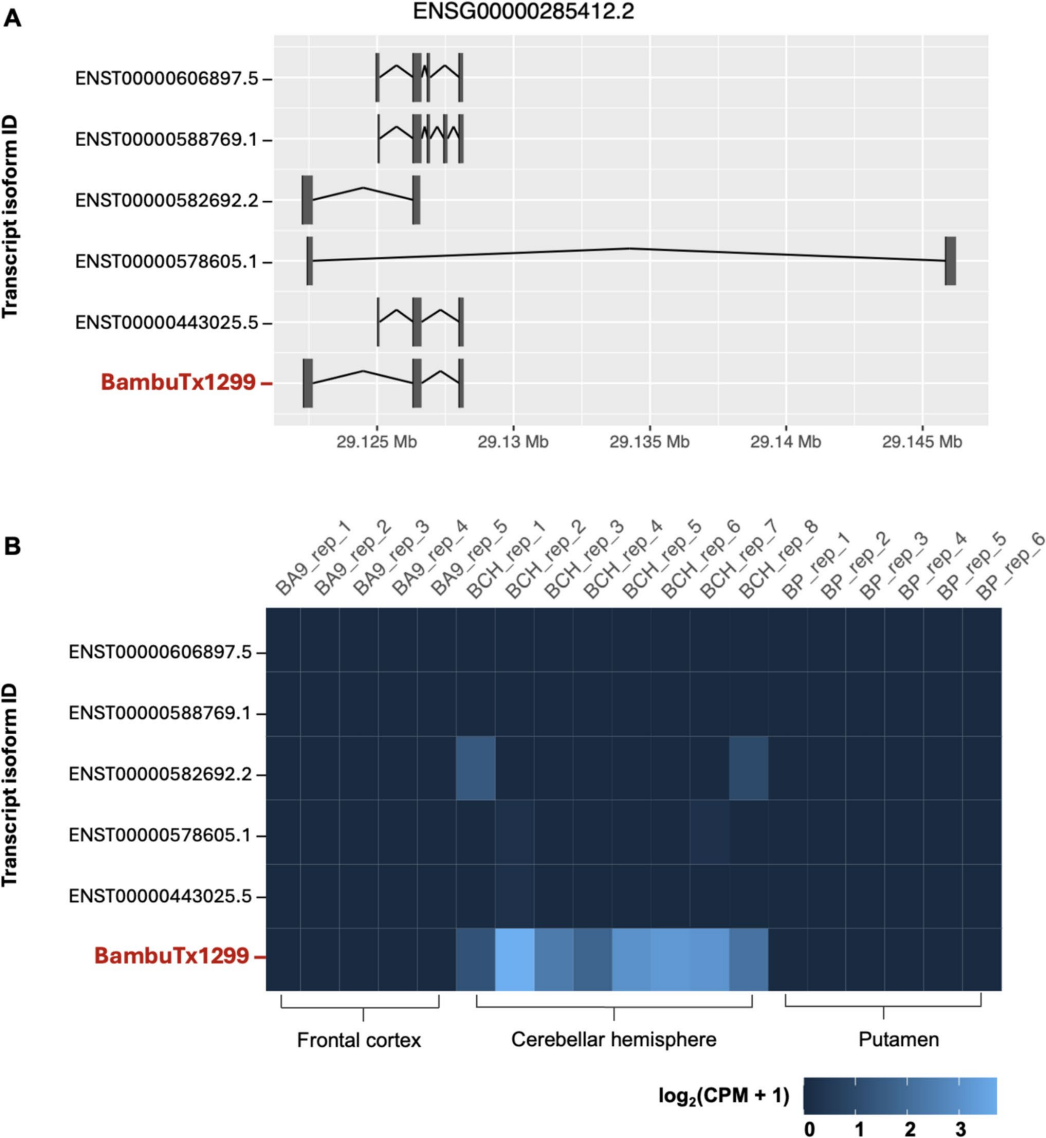
Visualisation of the long non-coding novel isoform BambuTx1299. **A** Visualisation of the structure of novel long non-coding isoform BambuTx1299 (red) compared against annotated isoforms of the same gene; *ENSG00000285412.2*. Shown are the genomic locations (x-axis) of all transcript isoforms’ (y-axis) exons (vertical lines) and splice junctions (horizontal joining bars) on the sense strand. The visualisation was generated with Bambu. v.3.6.0 in an R v4.40 environment. **B** A heatmap of the expression values of annotated and novel transcript isoforms for *ENSG00000285412.2*. Values are shown across all replicates (top x-axis) of each distinct brain region (bottom y-axis), coloured from low expression (dark blue) to high (light blue). Expression values are base-2 logarithmic transformations of normalised transcript expression levels (log_2_(CPM + 1)). The heatmap was generated with Bambu v.3.6.0 in an R v4.40 environment. Abbreviations: BA9, Brodmann area 9 (frontal cortex); BCH, cerebellar hemisphere; BP, putamen

The novel isoform uniquely expressed in the cerebellar hemisphere from the analysis set was found on the sense strand chr7:29,122,317–29,128,172 (Fig. 4A). This transcript (Bambu ID: BambuTx1299) is derived from the gene *ENSG00000285412.2* which has five annotated transcript isoforms, shown in Fig. 4A. BambuTx1299 is 783 nts in length, comprised of three exons, and is an unannotated combination of two known splice junctions. The mean CPM of BambuTx1299 across all replicates in the cerebellar hemisphere was 5.979 (SD, 3.812). The CPM of all other replicates in the putamen and frontal cortex was zero (Fig. 4B). Notably, the high expression is consistent across the different replicates, which is not seen for the annotated transcripts of *ENSG00000285412.2* (Fig. 4B).

**Fig. 5:**
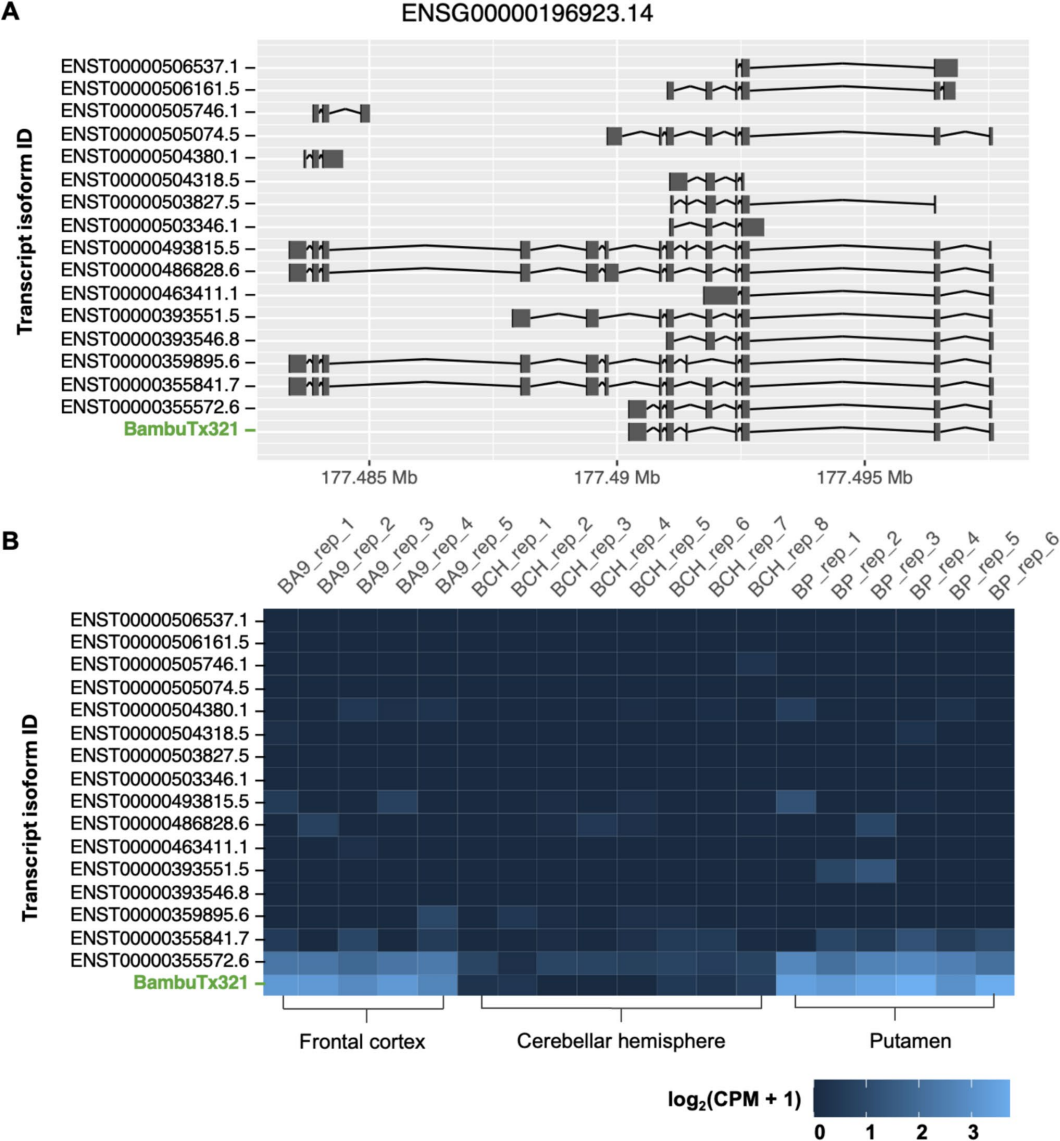
Visualisation of the novel protein-coding isoform BambuTx321. **A** Visualisation of the structure of novel protein-coding isoform BambuTx321 (green) compared against annotated isoforms of the same gene; *ENSG00000196923.14*. Shown are the genomic locations (x-axis) of all transcript isoforms’ (y-axis) exons (vertical lines) and splice junctions (horizontal joining bars) on the antisense strand. The visualisation was generated with Bambu. v.3.6.0 in an R v4.40 environment. **B** A heatmap of the expression values of annotated and novel transcript isoforms for *ENSG00000196923.14*. Values are shown across all replicates (top x-axis) of each distinct brain region (bottom y-axis), coloured from low expression (dark blue) to high (light blue). Expression values are base-2 logarithmic transformations of normalised transcript expression levels (log_2_(CPM + 1)). The heatmap was generated with Bambu. v.3.6.0 in an R v4.40 environment. Abbreviations: BA9, Brodmann area 9 (frontal cortex); BCH, cerebellar hemisphere; BP, putamen

BambuTx321 is a novel protein-coding transcript of the PDZ and LIM Domain 7 (*PDLIM7*) gene, which has 16 annotated transcripts. The transcript is 909 nts in length, located on the antisense strand chr5:177,490,246–177497607. The transcript is a novel combination of annotated splice junctions and contains eight exons (Fig. 5A). The CDS of BambuTx321 is 567 nts in length (chr5:177,496,512–177,490,497). The novel transcript is expressed at a higher degree in the putamen and frontal cortex in comparison to the cerebellar hemisphere (putamen – mean CPM, 22.116, SD, 6.148; frontal cortex – mean CPM, 14.974, SD, 3.488; cerebellar hemisphere – mean CPM, 0.29, SD, 0.278), resembling the expression of the annotated transcript *ENST00000355572.6* (Fig. 5B).

To validate both BambuTx321 and BambuTx1299, aligned short-read and long-read RNA-seq reads were visualised in Integrative Genomics Viewer v2.16.2 (Robinson et al. 2011) at their genomic coordinates in the cerebellar hemisphere and frontal cortex (respectively) (Figs. S1–2). There is a high coverage from both LRS and SRS at these two genomic locations which supports the correct mapping and structure of the novel isoforms (Figs. S1–2).

### Novel Isoform Protein-Coding Potential

**Fig. 6:**
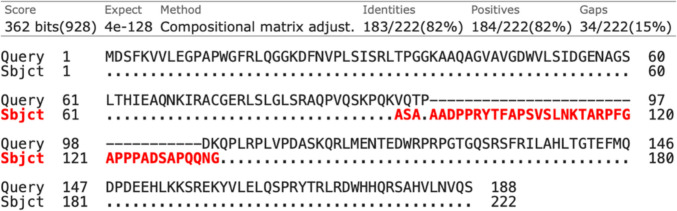
Alignment of the BambuTx321 ORF to the top BLAST match. A blastp search identified PDLIM7 isoform 4 as the top scoring human protein match for the ORF of BambuTx321. The BambuTx321’s ORF as the query sequence aligned to human PDLIM7 isoform 4 (NP_998801.1) is shown. Identities, or matches between the query and subject, are shown as dots whilst mismatches are indicated in red

Of the 170 novel isoforms, 104 contained a CDS and were determined to have protein-coding potential. The predicted ORFs of the 104 protein-coding transcripts were matched against sequences in the non-redundant protein sequence database with blastp. The first search identified 10 sequences with no significant similarity. Five of these sequences were ≤ 30 amino acids (aa) and hence not further investigated. The second search, filtering the non-redundant protein sequence database to human proteins, identified another two sequences that had no matches. Additionally, 14 sequences had a moderate to low homology with known human proteins, suggesting they are novel isoforms of known proteins. The top human protein match for BambuTx321 was the PDZ and LIM domain protein 7 (PDLIM7) isoform 4 (NP_998801.1) (percent identity, 82.43; E-value, 4e − 128; query cover, 100%) (Fig. 6).

**Fig. 7:**
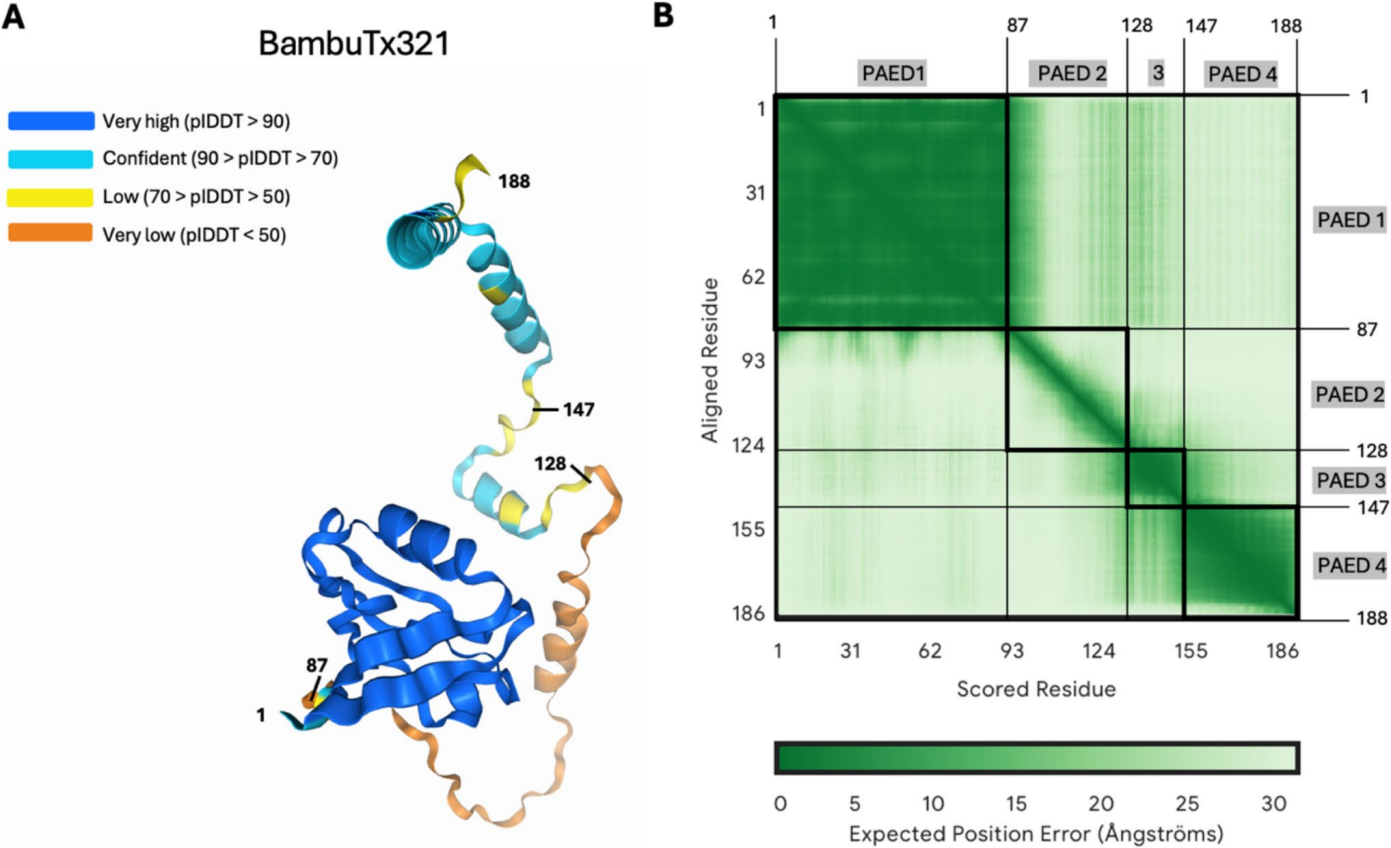
AlphaFold 3 structural prediction of novel transcripts ORF’s. The predicted protein structures of the ORF’s of novel protein-coding transcripts were generated with AlphaFold 3. **A** Shown are the plDDT score ranges for individual residues in the predicted protein structures. **B** The PAE scores, otherwise known as the expected position error, are shown for each residue pair in the structural predictions of BambuTx321. Abbreviations: PAE, predicted aligned error; PAED, predicted aligned error domain; plDDT, predicted local distance difference test

The predicted protein structure of BambuTx321’s ORF was poor (pTM = 0.46), particularly in the spatial arrangement of the four PAEDs, as seen by the white regions in the PAE plot (Fig. 7B). Additionally, PAED 2 was of very low confidence at the local residual level (plDDT < 50) (Fig. 7A) and domain structure level as indicated by the high PAE. Notably, the prediction of PAED 1 was of very high local confidence (plDDT > 90) and spatial arrangement, as indicated by the dark green colour (low PAE) of the entire domain (Fig. 7B). PAED 3 and 4 were also moderately confident at the local level (plDDT < 90) and domain structural level, indicated by the low PAE.

**Fig. 8:**
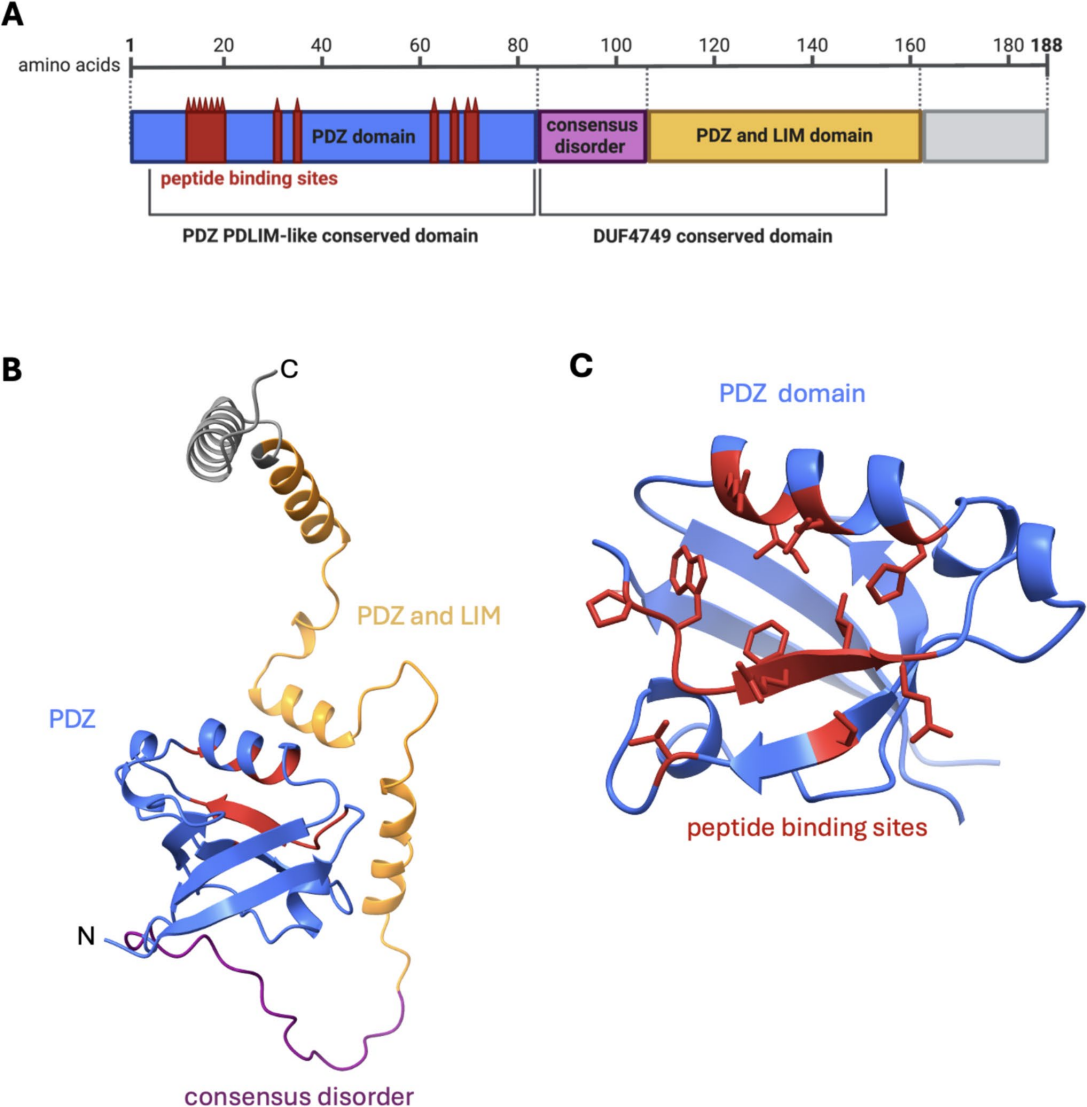
Domains and conserved regions of BambuTx321. **A** The domains, conserved regions, and conserved peptide binding sites of the BambuTx321 ORF identified by InterProScan and NCBI Conserved Domain Search. **B** The domains are indicated on the respective AlphaFold 3 predicted protein structure. **C** An enhanced view of the PDZ domain (blue) with peptide binding sites (red). Figures were generated with BioRender and ChimeraX

The ORF of BambuTx321 was uploaded to InterProScan and NCBI Conserved Domain Search for the identification of protein domains and conserved regions. The InterProScan search identified 13 protein domains, which have been summarised as a PDZ domain (1–84 aa), a consensus disorder domain (85–106 aa), and a PDZ and LIM domain (107–188 aa) (Fig. 8A). The PDZ signalling domain (cd00992) was also identified from five to 79 aa. This signalling domain features nine protein binding sites located at amino acid positions 13–16, 18, 66–67, and 70–71. BambuTx321 featured 75 conserved domain hits. The concise results identify PDZ PDLIM-like conserved domain (cd06753) from 5 to 83 aa (E-value 1.94e − 49) and the DUF4749 super family conserved domain (cl38478) from 84 to 155 (E-value 2.12e − 07), the latter of which is functionally uncharacterised. The conserved domain search also identified 13 peptide binding sites on the conserved PDZ PDLIM-like domain located at amino acid positions 13–19, 31, 34, 63, 67, and 70–71, which are indicated in red (Fig. 8). The three summarised protein domains and conserved peptide binding sites are indicated on the protein structural prediction of BambuTx321’s ORF (Fig. 8).

## Discussion

Alternative splicing (AS) can produce multiple RNA isoforms transcribed from the same gene. The scale and diversity of the human brain transcriptome are primarily attributed to the substantial occurrence of AS. This process, along with pervasive transcription across a wide variety of cell types, increases the magnitude and variety of protein-coding and non-coding RNAs from a single genome (Deveson et al. 2017). Thus, to identify and accurately quantify annotated and novel transcript isoforms, it is critical to produce reads that cover multiple splice junctions at high coverage for unambiguous assignment of reads to isoform (Aguiar et al. 2018). As LRS experiments have demonstrated the potential for novel isoform discovery and improved quantification (Chen et al. 2023; Cole et al. 2020; Dana et al. 2020; Fang et al. 2021; Gao et al. 2023; Zhang et al. 2023, 2020; Zhou et al. 2023), this study aims to explore and provide a more comprehensive understanding of the transcriptomic landscape of the human brain across the frontal cortex, cerebellar hemisphere, and putamen. It has been demonstrated in the literature that a significant proportion of probable novel isoforms are artifacts, which are caused by sequencing noise, RT-switching events, or limitations of the methodologies employed (Pardo-Palacios et al. 2024a, b). By applying ML refinements, rule-based filtering techniques, and incorporating findings across three different methods, a highly confident set of 170 unannotated RNA isoforms was discovered.

### Novel Isoform Discovery

The Long-read RNA-Seq Genome Annotation Assessment Project (LRGASP) Consortium recently evaluated computational methods for transcript identification and quantification which are suitable for long-read approaches (Pardo-Palacios et al. 2024a, b). The project compared the performance of 14 tools including Bambu, FLAIR, LyRiC, Isotools, StringTie2, Spectra, TALON, ISO_IB, FLAMES, IsoQuant, Mandalorion, NanoSim, RNA-Bloom, and rnaSPAdes (Bushmanova et al. 2019; Chen et al. 2023; Dana et al. 2020; Kovaka et al. 2019; Lienhard et al. 2023; Nip et al. 2020; Prjibelski et al. 2023; Silvia et al. 2023; Tang et al. 2020; Tian et al. 2021; Volden et al. 2023). The overall findings of the LRGASP proposed that for well-annotated organisms, Bambu, IsoQuant, and FLAIR were the strongest performing tools for isoform discovery and quantification. The consortium also recommended that utilising more than one tool can significantly improve the accuracy of novel isoform detection. Based on these findings, we demonstrated the ability to curate novel transcripts detected by FLAIR, Bambu, and IsoQuant. These isoforms were consistent in structure and expression across the three discovery methods and had passed post-assembly filtering and manual inspection. This was conducted to reduce the risk of further investigating false positive novel isoforms. In total, there were 170 isoforms in the analysis set, 104 of which were determined to have protein-coding potential. Most of the isoforms independent of their protein-coding potential were expressed to some degree in all three brain regions. Notably, seven novel isoforms were expressed in only the frontal cortex and putamen, which was reflective of the discerned similarity between the transcriptomic profiles of these two regions. The frontal cortex and cerebellar hemisphere each expressed a lncRNA with expressivity specific to their region, yet no novel isoforms in the analysis set were uniquely expressed in the putamen.

### Novel Long Non-coding RNA Isoform BambuTx1299

BambuTx1299 is a novel long non-coding isoform of the *ENSG00000285412* gene, which has not been previously described in the literature. The *ENSG00000285412* gene, also known as uncharacterised LOC128966558, is located on the forward strand of chromosome 7 and is antisense to the protein-coding carboxypeptidase vitellogenic-like (*CPVL*) gene (Mahoney et al. 2001). *CPVL* has two known antisense RNA: *CPVL* antisense RNA 1 (*CPVL*-AS1) and *CPVL* antisense RNA 2 (*CPVL*-AS2)*. CPVL*-AS1 and *CPVL*-AS2 are located on the forward strand and have one and four known transcripts respectively. LOC128966558 is also located on the sense strand and has five known isoforms which are associated with the lncRNA class. All known transcripts of LOC128966558 are undefined or have poorly described functions (Barshir et al. 2021). The transcripts, including BambuTx1299, are completely contained within the first intron of *CPVL.* Notably, although annotated isoforms of LOC128966558 are very lowly expressed in the cerebellar hemisphere, BambuTx1299 shows moderate to high expression across all replicates (Fig. 4B). Additionally, the novel isoform was only detected in the cerebellar hemisphere, which could help elucidate its function. *CPVL* is also expressed at an elevated level in the cerebellar hemisphere and cerebellum in comparison to other regions of the brain (GTEx Consortium 2013, 2020). The function and role of LOC128966558 are yet to be addressed in the literature, and no associations with disease or pathology has been described. Considering the *CPVL* gene has known associated antisense RNAs, LOC128966558 may also be an antisense RNA gene of *CPVL.* Taking into consideration that the transcripts of LOC128966558 are in antisense orientation to *CPVL*, which is a protein-coding gene, they may function to regulate *CPVL*’s expression. This may occur through the inhibition or upregulation of transcription in tissues where they are co-expressed, such as the cerebellar hemisphere (Santos et al. 2022; Zhang et al. 2019). However, this is a naïve assumption and the functions of both *CPVL* and *ENSG00000285412* must be meticulously studied before trying to infer the biological roles and interactions of novel isoform BambuTx1299 (Mattick et al. 2023; Navandar et al. 2024).

### Novel Protein-Coding Isoform BambuTx321

BambuTx321 is a novel mRNA transcribed from *PDLIM7*, which exhibited an elevated expression in the putamen and frontal cortex, reflective of the expression of the annotated transcript *ENST00000355572.6* (Fig. 5B). RNA expression of other known PDLIM7 isoforms is elevated in several organs and tissues, including skeletal muscle, the gastrointestinal tract, and gonadal tissues, as well as a moderate expression in other tissues such as the brain (Uhlén et al. 2015). Unsurprisingly, the top human protein match for ORF of BambuTx321 was of PDLIM7 isoform 4 (Fig. 6). This protein is translated from *ENST00000355572.6* which only differs from BambuTx321 by the fifth exon (Fig. 5A). The predicted protein structure of BambuTx321’s ORF displayed poor confidence (pTM = 0.46); however, PAED 1 was of very high local confidence (plDDT > 90) and spatial arrangement. This region corresponds to the PDZ domain, containing the conserved peptide binding sites (Fig. 8). Multiple isoforms of PDZ-LIM proteins have been explored in the literature, as abundant AS of their precursor genes results in numerous combinations and conformations of the PDZ and PDZ-LIM protein domains (te Velthuis & Bagowski 2007). It seems that BambuTx321 could potentially encode a truncated protein, caused by the skipping of exon 5 (relative to antisense transcript *ENST00000355572.6*), which does not impact the conserved PDZ domain nor peptide binding sites. The family of PDZ-LIM proteins has seven members (PDLIM1-7), which mediate the organisation of actin cytoskeleton by their ability to bind to actin (Healy & Collins 2023). The proteins of this family are involved in a variety of signal transduction pathways and other biological roles, such as organ development, intracellular signalling, neuronal signalling, and oncogenesis (Ponting et al. 1997; Rood et al. 2023; te Velthuis & Bagowski 2007). In brain and epithelial tissues, the PDZ domain has demonstrated roles in polarised protein localisation through its protein interactions (Rongo 2001). This domain, which was conserved in BambuTx321, binds to specific peptide sequences (PDZ binding motifs) which are typically located at the C-terminal end of their partner proteins (Lee & Zheng 2010). A recent study demonstrated that PDLIM7 protein associates with synaptopodin, which is specifically expressed in the spine apparatus, an area of the neuronal smooth endoplasmic reticulum (ER) located in dendritic spines (Falahati et al. 2022). In cultured hippocampal neurons of mice, PDLIM7 colocalised with and exhibited a similar expression to that of synaptopodin which suggests there is a functional relationship between the two proteins in dendritic spine physiology (Falahati et al. 2022). Additionally, a genome-wide association study on educational attainment identified an association between PDLIM7 with mathematical ability, which in conjunction with its function in dentritic spines suggests its involvement in learning and cognitive performance (Lee et al. 2018). Considering this, BambuTx321 is suspected to have diverse functional roles in the human brain which largely reflect those of PDLIM7 isoform 4.

### Limitations and Future Directions

Nanopore sequencing was performed on total RNA which was isolated from bulk tissue extractions from the putamen, frontal cortex, and cerebellar hemisphere. Therefore, for the analysis of novel isoforms with brain region–specific expression, such as BambuTx1299, it cannot be determined whether the isoforms are specific to the entire region or to a particular cell type. Further experiments utilising single-cell sequencing technologies or fluorescence in situ hybridisation could be conducted to determine the cell specificity and localisation of these novel isoforms, which could also aid in elucidating their functions (Falahati et al. 2022). As only three brain regions were analysed in this study, it is unknown whether these isoforms are expressed in other brain regions or in tissues of other systems. This could be quite easily determined by using the analytical pipeline on an expanded scope of samples, which would almost certainly uncover more unannotated isoforms for characterisation. Furthermore, future analyses may identify novel isoforms and transcriptomic alterations associated with various brain diseases and disorders. Such isoforms may represent candidates for the development of targeted therapeutic strategies, such as antisense oligonucleotides (ASOs), which can be designed to modulate gene expression at the RNA level by degrading targeted mRNAs. Additionally, the identification of these novel isoforms may also act as diagnostic biomarkers, facilitating earlier detection and precise monitoring of disease progression.

Across the three different discovery methods, 170 novel isoforms were identified. Rule-based and manual filtering was also performed to reduce the potential of analysing false positive novel isoforms. However, these novel isoforms are yet to be experimentally validated. Thus, it is suggested that further investigation into the legitimacy of these isoforms is to be undertaken. This could be conducted in numerous manners, such as the targeting of regions specific to the novel isoform with primers for targeted PCR amplification and sequencing. To confirm the existence of protein products and to find evidence supporting the translation of novel mRNA isoforms, matching peptide sequences could be found in mass spectrometry data (Heberle et al. 2023). It is important to note that evidence for the alternatively spliced proteins is generally very low (Pozo et al. 2021).

Despite the integrity of computational ab initio structural predictions, the protein structures are predictions, and the true structure of these proteins is likely to be different at low-confidence regions. Therefore, to validate the protein structures at atomic resolution, techniques such as X-ray crystallography, nuclear magnetic resonance (NMR), or single-particle cryo-electron microscopy (cryo-EM) should be utilised for more confident structure determination. For the protein encoded by BambuTx321, cryo-EM in combination with tomography would be suitable for the study and visualisation of protein complexes in neurons, such as with synaptopodin (Martinez-Sanchez et al. 2020).

## Concluding Remarks

Overall, this study has developed a robust analytical pipeline enabling the discovery of previously obscured segments of the non-coding and protein-coding transcriptome. Furthermore, by identifying novel protein-coding isoforms, this pipeline can facilitate the discovery of unexplored portions of the proteome. As a result, new elements in biological pathways and the revelation of novel therapeutic and diagnostic markers can be achieved. This is especially true for studies that would adopt comparative expression analyses of diseased and healthy conditions. By re-exploring existing datasets in this study, 170 new non-coding and protein-coding isoforms have been identified in various regions of the human brain, contributing to and enhancing the quality of reference genome repositories such as NCBI and Ensembl.

## Supplementary Information

Below is the link to the electronic supplementary material.

Supplementary file1 (XLSX 15103 KB)

Supplementary file2 (DOCX 460 KB)

## Data Availability

No datasets were generated or analysed during the current study.

## Code Availability

All code and scripts required to reproduce the analyses and figures in this manuscript is available on GitHub (https://github.com/janitz-lab/novel-isoform-discovery).
