# Supplementary material for: Discovery of Novel Protein-Coding and Long Non-coding Transcripts in Distinct Regions of the Human Brain
Source: J Mol Neurosci. 2025 Mar 6;75(1):30. doi: 10.1007/s12031-025-02316-9 (PMC11885362; doi:10.1007/s12031-025-02316-9)
Supplement: Supplementary file 2 — Supplementary file2 (DOCX 460 KB) [file 12031_2025_2316_MOESM2_ESM.docx]

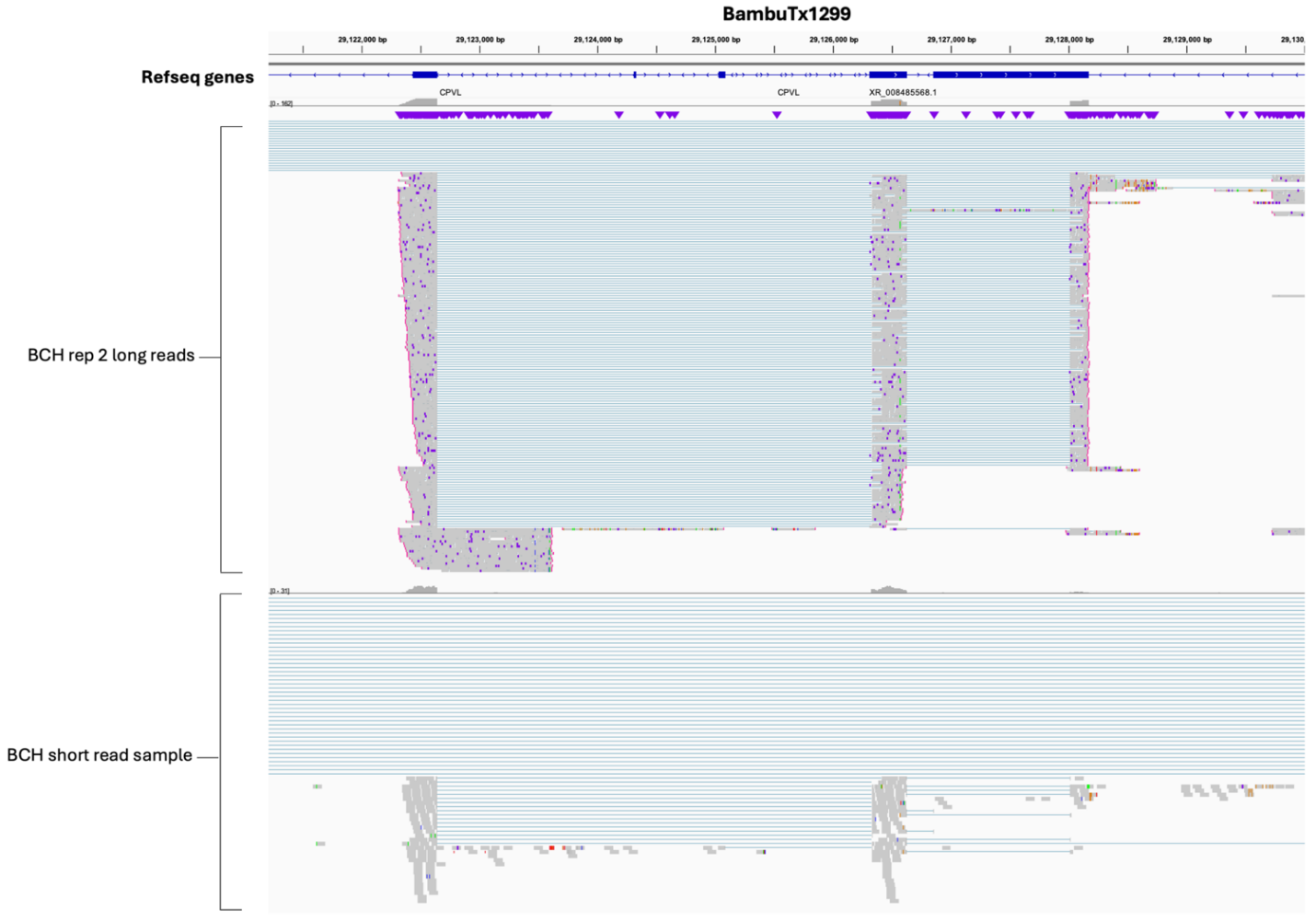


**Figure S1.** **Aligned sequencing reads near CPVL in the cerebellar hemisphere.** Visualisation of aligned long-read and short-read RNA-sequencing reads in Interactive Genomics Viewer (IGV). Long reads were aligned using a 2-pass splice-aware approach with 2passtools and Minimap2 (see main text) and the short-read RNA-seq data was aligned with STAR. All reads are shown at the genomic location of ENSG00000285412.2 (XR_008485568.1) which is antisense to the carboxypeptidase vitellogenic like (CPVL) gene. Abbreviations: BCH – Cerebellar hemisphere; Refseq – NCBI Reference Sequence Database.


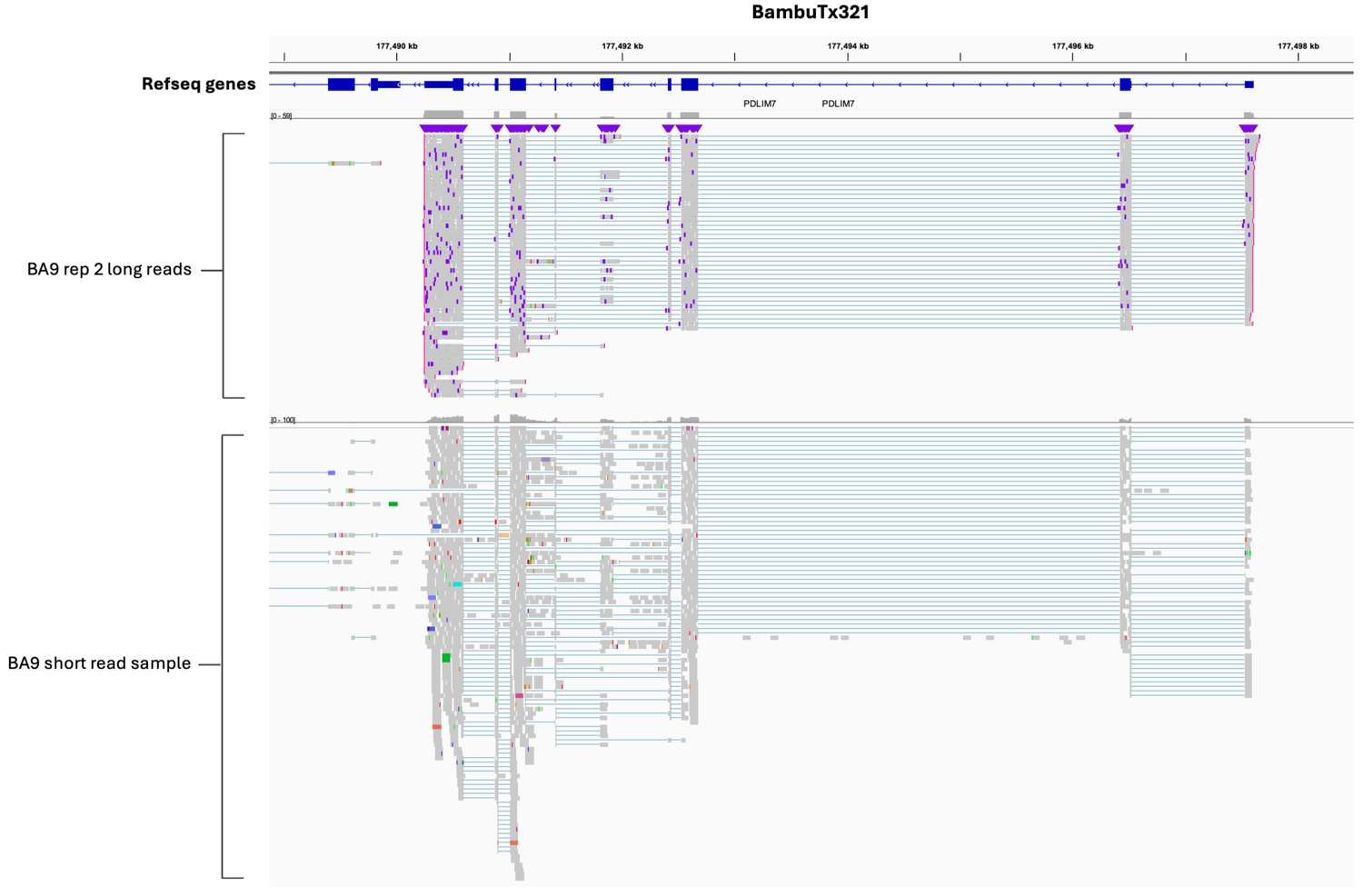


**Figure S2.** **Aligned sequencing reads near PDLIM7 in the frontal cortex.** Visualisation of aligned long-read and short-read RNA-sequencing reads in Interactive Genomics Viewer (IGV). Long reads were aligned using a 2-pass splice-aware approach with 2passtools and Minimap2 (see main text) and are visualised alongside the STAR aligned short-read RNA-seq data. Reads are shown at the genomic location of PDLIM7. Abbreviations: BA9 – Brodmann area 9 (frontal cortex); Refseq – NCBI Reference Sequence Database.
